# Feasibility of Adaptive MR-guided Stereotactic Body Radiotherapy (SBRT) of Lung Tumors

**DOI:** 10.7759/cureus.2423

**Published:** 2018-04-04

**Authors:** Kyle R Padgett, Garrett N Simpson, Ricardo Llorente, Michael A Samuels, Nesrin Dogan

**Affiliations:** 1 Department of Radiation Oncology, Sylvester Comprehensive Cancer Center, Miller School of Medicine, University of Miami, Miami, USA

**Keywords:** radiation therapy, smart, magnetic resonance imaging (mri), magnetic resonance-guided radiation therapy (mrgrt), lung stereotactic body radiotherapy (sbrt)

## Abstract

Online adaptive radiotherapy (ART) with frequent imaging has the potential to improve dosimetric accuracy by accounting for anatomical and functional changes during the course of radiotherapy. Presented are three interesting cases that provide an assessment of online adaptive magnetic resonance-guided radiotherapy (MRgRT) for lung stereotactic body radiotherapy (SBRT).

The study includes three lung SBRT cases, treated on an MRgRT system where MR images were acquired for planning and prior to each treatment fraction. Prescription dose ranged from 48 to 50 Gy in four to five fractions, normalized to where 95% of the planning target volume (PTV) was covered by 100% of the prescription dose. The process begins with the gross tumor volume (GTV), PTV, spinal cord, lungs, heart, and esophagus being delineated on the planning MRI. The treatment plan was then generated using a step-and-shoot intensity modulated radiotherapy (IMRT) technique, which utilized a Monte Carlo dose calculation. Next, the target and organs at risk (OAR) contours from the planning MRI were deformably propagated to the daily setup MRIs. These deformed contours were reviewed and modified by the physician. To determine the efficacy of ART, two different strategies were explored: 1) Calculating the plan created for the planning MR on each fraction setup MR dataset (Non-Adapt) and 2) creating a new optimized IMRT plan on the fraction setup MR dataset (FxAdapt). The treatment plans from both strategies were compared using the clinical dose-volume constraints.

PTV coverage constraints were not met for 33% Non-Adapt fractions; all FxAdapt fractions met this constraint. Eighty-eight percent of all OAR constraints studied were better on FxAdapt plans, while 12% of OAR constraints were superior on Non-Adapt fractions. The OAR that garnered the largest benefit would be the uninvolved lung, with superior sparing in 92% of the FxAdapt studied. Similar, but less pronounced, benefits from adaptive planning were experienced for the spinal cord, chest wall, and esophagus.

Online adaptive MR-guided lung SBRT can provide better target conformality and homogeneity and OAR sparing compared with non-adaptive SBRT in selected cases. Conversely, if the PTV isn’t adjacent to multiple OARs, then the benefit from ART may be limited. Further studies, which incorporate a larger cohort of patients with uniform prescriptions, are needed to thoroughly evaluate the benefits of daily online ART during MRgRT.

## Introduction

Lung stereotactic body radiotherapy (SBRT) has proven to be effective in the local control of lung tumors [1]. However, significant motion of the lesion, motion of the organs at risk (OAR), as well as their proximity to the lesion and alterations to the anatomy, and motion characteristics throughout the course of treatment make radiation therapy of lung tumors challenging. Online adaptive radiotherapy (ART) has recently become available with the arrival of MR-guided radiotherapy (MRgRT) devices, which allow for both high-resolution three-dimensional (3D) magnetic resonance imaging (MRI) scans, allowing for accurate daily visualization of the disease and OARs, as well as real-time cine imaging to accurately gate a radiation treatment in the proper phase of the respiration cycle. Utilizing MR-guided ART has the potential to improve target coverage and to provide better OAR sparing by using the daily 3D MRI to account for anatomical and functional changes and adapt the plan to deliver the optimal treatment for that day [2-4].

The benefit of online ART using MRgRT systems has been demonstrated for several types of cancer but only limited experience with lung SBRT ART has been published [2]. Work by Henke et al. investigated the potential benefits of the simulated online ART MRgRT for five central thorax and five non-liver abdominal cases. For thoracic cases, the results revealed that common OAR violations were the trachea and esophagus. Increased PTV coverage in 21 of 30 fractions was obtained. Furthermore, additional OAR sparing was achieved in 63% fractions while escalating the dose to PTV.

Presented are three interesting cases that provide an assessment of online adaptive MR-guided radiotherapy (MRgRT) for lung SBRT with the purpose of beginning to determine under what circumstances are there significant advantages to ART and, just as crucial, under what circumstances it does not provide a measurable benefit.

## Case presentation

Patient population

Three diverse lung SBRT patients treated at our clinic are presented to demonstrate in which circumstances daily online ART will provide a benefit. Case #1 is a 66-year-old male with a T3N1M1a adenocarcinoma of the lung; M1a disease was designated due to a solitary tumor in the opposite lung. He received 50 Gy in four fractions to the central lesion in the right lung. Case #2 is an 87-year-old female with a cT1bN0M0 lung adenocarcinoma. She received 50 Gy in five fractions to the peripheral lesion in the right lung. Case #3 is a 70-year-old male with an adenocarcinoma of the pancreas metastatic to the lung. He received 48 Gy in four fractions to the peripheral lesion in the left lung. A summary of the clinical characteristics for all cases is presented in Table 1.

**Table 1:** Patient Clinical Characteristics Fx(s): fraction(s); GTV: gross tumor volume ; PBT: proximal bronchial tree;  PTV: planning target volume

	Case #1	Case #2	Case #3
Age (years)	66	87	69
Prescribed Dose # of Fxs	50 Gy in 4 fx	50 Gy in 5 fx	48 Gy in 4 fx
Primary Disease	Lung Adenocarcinoma	Lung Adenocarcinoma	Pancreatic Adenocarcinoma
Tumor Staging	T3N1M1a	T1bN0M0	Metastatic
Location within the Lung	Central	Peripheral	Peripheral
PTV Coverage Constraint	> 95% @ 50.0 Gy	> 95% @ 50.0 Gy	> 95% @ 48.0 Gy
Pulmonary Constraint #1	< 1500 cc @ 11.6 Gy (Both Lungs – GTV)	< 1500 cc @ 12.5 Gy (Both Lungs – GTV)	< 4 cc @ 15.6 Gy PBT
Pulmonary Constraint #2	N/A	< 1000 cc @ 13.5Gy (Both Lungs – GTV)	N/A
Spinal Cord Constraint #1	Max Dose < 26.0 Gy	Max Dose < 26.0 Gy	< 0.35cc @ 20.8 Gy
Spinal Cord Constraint #2	< 0.35 cc @ 20.8 Gy	< 0.25 cc @ 22.5 Gy	< 1.2 cc @ 13.6 Gy
Spinal Cord Constraint #3	< 1.2 cc @ 13.6 Gy	< 0.5 cc @ 13.5 Gy	N/A
Chest Wall / Ribs	N/A	Chest Wall < 30 cc @ 40.0 Gy	Ribs < 1 cc @ 32.0 Gy
Esophagus	< 5 cc @ 18.8 Gy	< 5 cc @ 27.5 Gy	< 5 cc @ 18.8 Gy
Heart	< 15 cc @ 28.0 Gy	< 15 cc @ 32.0 Gy	< 15 cc @ 28.0 Gy
GTV Volume Initial (cc)	6.91	41.39	1.91
GTV Volume Final (cc)	8.23	36.97	1.64
GTV Volume % Change	+19%	-11%	-14%

MRgRT system

The MRgRT system by Viewray™ (ViewRay, Inc., Cleveland, OH, USA) has been described previously but briefly consists of a 0.35-Tesla split-bore design MRI scanner with three Cobalt-60 sources separated by 120 degrees mounted on a ring gantry [5]. Each of the three Cobalt-60 sources utilized a dual-focused multileaf collimator (MLC) with 1.05 cm leaf thickness; when all three heads are treating simultaneously, the nominal dose rate is 550 cGy/minute. The real-time MRI capabilities of the system allow for soft tissue imaging throughout radiation therapy (RT) delivery and the IMRT delivery is achieved by a “step-and-shoot” technique. The MRI system is a vertically gapped (double donut) horizontal solenoidal superconducting 0.35 T whole-body MRI. The split gradient coil has an inner diameter of 80 cm with a maximum gradient strength of 18 mT/m and a maximum slew rate of 200 T/m/s.

Contouring and treatment planning

For all patients, a deep inspiration planning MRI and a planning computed tomography (CT) were collected at the time of simulation where the MRI was utilized for treatment planning. The electron density was deformably propagated from the CT to the planning MRI and was reviewed by a medical physicist. The treatment planning targets and OARs were contoured by an experienced radiation oncologist and a GTV to PTV expansion of either 3 mm or 6 mm was utilized for planning. Step-and-shoot IMRT plans were created using a single-isocenter, typically seven or nine beam groups (19 - 25 beam angles), avoiding beams that pass through the couch edges. Monte Carlo dose calculations with a 3 mm dose grid were employed by the treatment planning system (TPS), and all plans included the influence of the 0.35 T magnetic field on the radiation dose calculation. Real-time MRI-guided gating was utilized during treatment delivery to ensure that the treatment planning target was within the treatment field.

For all FxAdapt and Non-Adapt fractional plans, the electron density was deformably propagated from the planning CT to the daily MRI. Following this, the GTV contour was rigidly propagated and OAR contours were deformably propagated to the daily setup MRI. An experienced radiation oncologist would edit the contours as needed and then the PTV structures and other optimization structures would be created.

The Non-Adapt fractional plans were created by calculating the original treatment plan on the daily setup MRIs. The isocenter of the plan was placed on the daily setup MRI by rigidly registering the daily setup MRI to the planning MRI by focusing on GTV alignment between the two datasets. Once the contours had been finalized, the original plan was calculated on the daily MRI dataset. The FxAdapt plans were created by re-optimizing the original treatment plan on the daily setup MRI, with the daily electron density map and the daily treatment targets and OARs. The same optimization parameters were utilized for the FxAdapt plans as the initial plan, and following the re-optimization, the FxAdapt was normalized so that 100% of the prescribed dose covered 95% of the PTV.

## Discussion

Case 1

**Figure 1:**
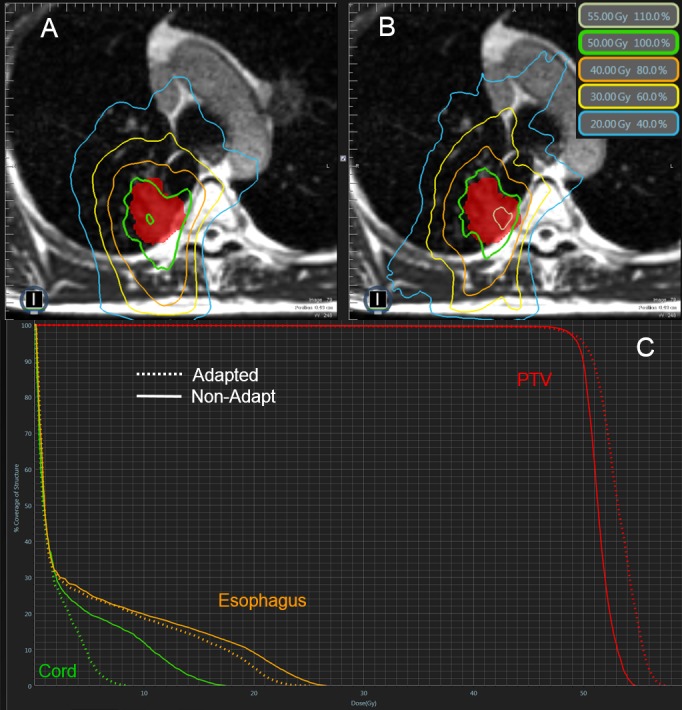
Review of Case 1 Example of a single Non-Adapt and FxAdapt fraction for Case 1. A) Non-Adapt isodose lines, B) FxAdapt isodose lines, C) dose-volume histogram (DVH) for both Non-Adapt (solid line) and FxAdapt (dashed line). Note the conformality of the FxAdapt example as compared to the Non-Adapt example and note the better target coverage for FxAdapt. Also, FxAdapt has a reduced dose to the spinal cord.

Case 1 presents a 7 cc central lesion that was adjacent to both the spinal cord and the esophagus (Figure 1). The prescription dose was to deliver 50 Gy in four fractions where 95% of the PTV was to receive 100% of the dose and the primary organs at risk for this case consisted of the lungs minus PTV (lungs-PTV), spinal cord, and esophagus. The initial plan satisfied all constraints, including PTV coverage constraints, but the Non-Adapt fractions struggled to achieve PTV coverage with two of the four fractions being below 95% coverage (89% and 94%). The FxAdapt fractions all achieved exactly 95% coverage; this is to be expected because the reoptimized plans were all renormalized to 95% coverage. The lung PTV constraint was easily achieved for both Non-Adapt and FxAdapt fractions, less than 1,500 cc at 11.6 Gy, but the FxAdapt fractions irradiated significantly less lung tissue, 516 cc vs 704 cc, respectively. The max dose limit to the spinal cord, 26 Gy, was achieved for all Non-Adapt and FxAdapt fractions with the FxAdapt fractions delivering significantly less dose, 12.1 Gy vs 19.2 Gy, respectively. The Non-Adapt fractions struggled to achieve constraint tolerance for the esophagus with one of the fractions exceeding tolerance, less than 5 cc at 18.8 Gy, whereas the Non-Adapt averaged 5.1 cc and the FxAdapt fractions all easily achieved tolerance and averaged on 1.9 cc at 18.8 Gy.

Case 2

**Figure 2:**
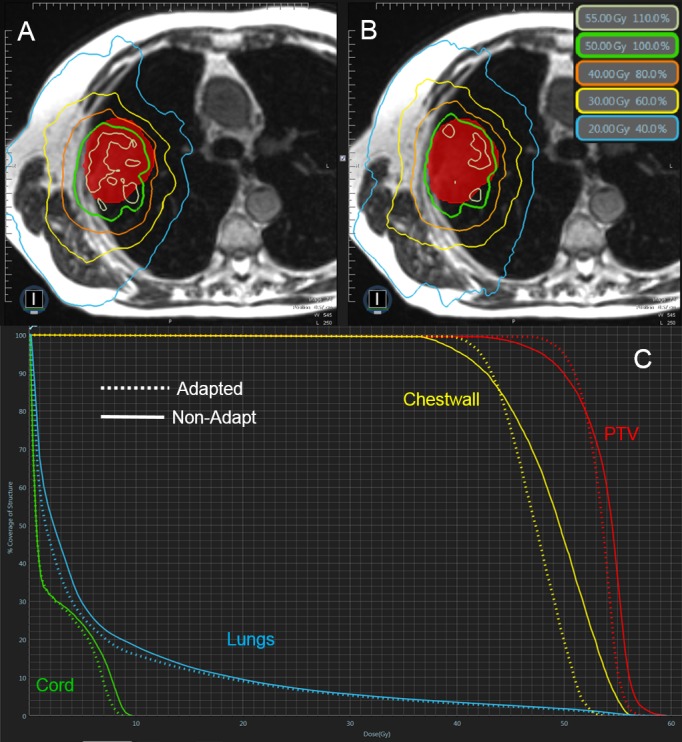
Review of Case 2 Example of a single Non-Adapt and FxAdapt fraction for Case 2. A) Non-Adapt isodose lines, B) FxAdapt isodose lines, C) dose-volume histogram (DVH) for both Non-Adapt (solid line) and FxAdapt (dashed line). Similar to Case 1, the conformality of the FxAdapt is superior to that of the Non-Adapt example. Also, the target coverage is superior to the Non-Adapt as well. There is also a reduced dose to the chest wall.

Case 2 presents a 41 cc peripheral lesion that was adjacent to the chest wall. The prescription was to deliver 50 Gy in five fractions where 95% of the PTV was to receive 100% of the dose (Figure 2). The constraints of interest consisted of lungs-PTV, chest wall, and maximum point dose (Dmax). Similar to Case 1, the initial plan satisfied all constraints and PTV coverage was not much of an issue for the Non-Adapt fractions with four of five achieving 95% coverage; all FxAdapt fractions achieved coverage constraints. The Non-Adapt fractions did struggle to achieve Dmax constraints with all five fractions exceeding the 57.5 Gy constraint, averaging 59.7 Gy. The FxAdapt fractions fared better with three of the five achieving the constraint and averaging 57.7 Gy. Similar to Case 1, the lungs-PTV constraint was easily achieved for both Non-Adapt and FxAdapt fractions, less than 1,000 cc at 13.5 Gy, with similar irradiated volumes at this dose level, 552 cc Non-Adapt and 509 cc FxAdapt. The constraint most difficult to achieve was to irradiate less than 30 cc of the chest wall with a dose of 40 Gy or higher. All five Non-Adapt fractions failed this constraint with four of the five failing for the FxAdapt fractions. The FxAdapt fractions did irradiate less volume than the Non-Adapt at this dose level, 32 cc vs 37 cc, respectively.

Case 3

**Figure 3:**
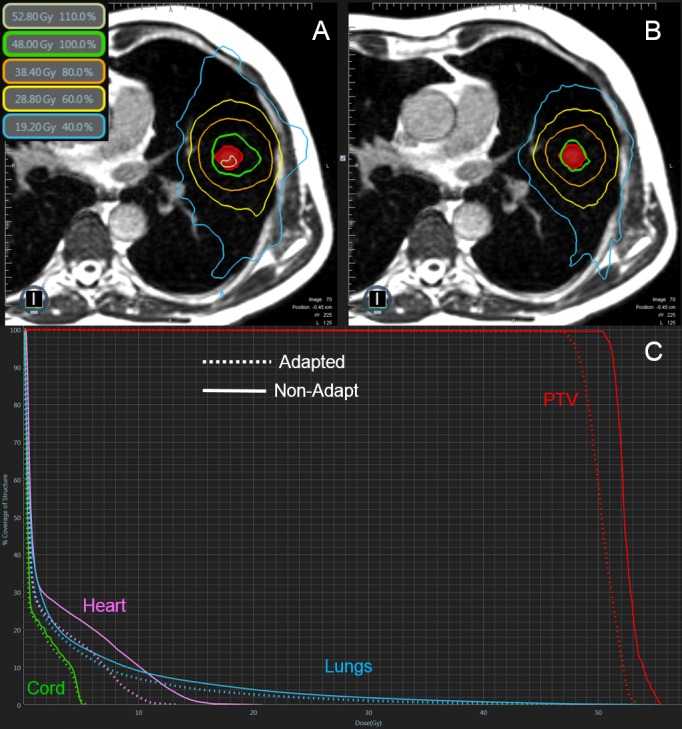
Review of Case 3 Example of a single Non-Adapt and FxAdapt fraction for Case 3. A) Non-Adapt isodose lines, B) FxAdapt isodose lines, C) dose-volume histogram (DVH) for both Non-Adapt (solid line) and FxAdapt (dashed line). Again, the conformality of the FxAdapt is superior to the Non-Adapt. Also, the FxAdapt is a cooler plan, with the Non-Adapt plan exceeding the prescribed dose.

Case 3 was different than the other two examples in that it was a smaller (2 cc) peripheral lesion that was not adjacent to another OAR other than the proximal bronchial tree (PBT). The prescription dose was to deliver 48 Gy in four fractions where 95% of the PTV was to receive 100% of the prescribed dose. The initial plan, the Non-Adapt, and FxAdapt plans all satisfied the PTV coverage constraints. The FxAdapt fractions were slightly cooler (Dmax average 55.0 Gy) than the Non-Adapt fractions (Dmax average 53.5 Gy). Similarly, the max dose constraint of 39 Gy to the PBT was achieved for all Non-Adapt (average 16.5 Gy) and FxAdapt fractions (14.4 Gy) (Figure 3).

Study limitations

There are several limitations in our study, namely, the small population size, diverse prescription doses, variable OAR constraints, and diverse tumor characteristics (such as varied histology, staging, and diverse locations within the lung). Additionally, Linac-based MRgRT systems have become available which have significant dosimetric advantages over the Cobalt-60-based MRgRT system used in this study. While the dosimetric properties of a Linac-based system are superior, the relative benefits one experiences from ART strategies should be independent between the two modalities. Therefore, our experiences should translate to those institutions that have access to the Linac-based MRgRT systems. To fully answer the question as to how much of a benefit MR-guided ART provides to lung SBRT patients, a more comprehensive study should be designed. It is our hope that the interesting cases presented here will help enlighten those that wish to treat these complicated cases and those that wish to design future studies.

## Conclusions

For certain lung SBRT patients, ART strategies have significant benefits when using MRgRT systems while others have more subdued or negligible benefits. From these few examples, having several OARs adjacent to the PTV is the strongest indicator of deriving a benefit from ART. In these cases, only slight changes in volumes or relative positions between the PTV and the OARs resulted in significant differences in both PTV coverage and OAR dose. In cases where the PTV coverage and constraints can be met without adaptation, further OAR sparing is a benefit of ART and potentially allows for dose escalation strategies. While our findings are consistent with the limited publications in this area, the work presented here focuses on lung tumors. Nevertheless, from our work and previously published studies, there is a consistent message that ART techniques combined with MRI guidance often achieves better PTV coverage and OAR sparing. In essence, online ART using MRgRT has the potential to provide better target conformity and OAR sparing for lung SBRT treatments allowing for dose escalation. Future studies are needed to confirm and characterize these potential benefits.
